# *Helleborus odorus* subsp. *cyclophyllus*: An Unexploited Source of Antioxidant, Antimicrobial, and Cytotoxic Bioactivity

**DOI:** 10.3390/biology15110852

**Published:** 2026-05-29

**Authors:** Panagiotis Sarametidis, Evgenia-Anna Papadopoulou, Panagiotis Katsoris, Konstantinos A. Aliferis, Keith D. Rochfort, Konstantinos Grintzalis

**Affiliations:** 1 School of Biotechnology, Dublin City University, D09 N920 Dublin, Ireland; panagiotis.sarametidis2@mail.dcu.ie (P.S.); keith.rochfort@dcu.ie (K.D.R.); 2 Department of Biology, University of Patras, 265 04 Patras, Greece; katsopan@upatras.gr; 3 Laboratory of Pesticide Science, Agricultural University of Athens, Iera Odos 75, 118 55 Athens, Greece; evina.papadopoulou@gmail.com (E.-A.P.); konstantinos.aliferis@aua.gr (K.A.A.)

**Keywords:** antibacterial, antioxidants, cytotoxic, *Helleborus* sp., human aortic endothelial cells, metabolomics, reactive oxygen species

## Abstract

*Helleborus* sp. are small, perennial herbs with a wide distribution across central and southern Europe and Asia. Historically, extracts of these plants have been used in remedies exhibiting cathartic and anthelmintic effects; however, their adverse and toxic potential have also been reported. As such, *Helleborus* extracts have been trialled as alternative approaches to cancer treatment in rare cases. In this study, a detailed phytochemical characterisation of root extracts from *Helleborus odorus* subsp. *cyclophyllus* was performed in parallel with an in vitro assessment of their cytotoxicity and effect on cell phenotype. GC/EI/MS metabolite profiling revealed the complexity of the extract, which exhibited a high relative content of fatty acids (17.98%), with the most abundant group being the unsaturated fatty acids, followed by saturated and hydroxy fatty acids. Bioassays indicated the extract had antioxidant capabilities, while in vitro analysis demonstrated the extract induced a pro-oxidant phenotype that reduced cell viability, inhibited growth in bacterial (*E. coli*) and mammalian (human aortic endothelial cells) cell types, and exacerbated pro-inflammatory indices. *Helleborus odorus* subsp. *cyclophyllus* contains bioactive molecules that elicit a number of cellular responses, and the therapeutic potential of these molecules warrants further investigation.

## Simple Summary

In the present day, plant-derived remedies are routinely used within traditional medicine. Furthermore, many commercially available compounds were initially isolated from plant sources. Hellebores are plants commonly found in Europe, and research conducted on their extracts has indicated inherent physiological effects on cells of the immune and vascular systems, amongst others. In this study, we characterised the chemical composition and antioxidant properties of the extract of the root from *Helleborus* sp. Following this, we assessed its impact on toxicity, oxidative stress, and angiogenesis using a human aortic endothelial cell model. Combining metabolomics with in vitro assays, our study aims to identify targets and therapies in relation to unexplored sources from hellebores.

## 1. Introduction

The interest in the therapeutic potential of bioactive molecules derived from medicinal plants has increased significantly over recent years. There are numerous references in the literature to the diverse properties of plant extracts in the context of health and disease, with many demonstrating their therapeutic effects through antioxidant, antibacterial, and antiviral means [1,2]. Moreover, individuals are less likely to develop resistance to plant-derived medicines compared to conventional drugs. As such, in line with the increasing interest in plant-derived alternative medicines, a correlative increase in the exploitation and adoption of plant extracts in health sciences has been observed, with many citing their use to treat infections, cancer, and circulatory disease.

*Helleborus* sp. belongs to the *Ranunculaceae* family and includes small, perennial herbs commonly found in central and southern Europe and Asia. Their distribution in Europe has elevated their significance in therapeutic remedies since antiquity [3]. In the literature, *Helleborus niger* is the most widely reported species used in a medicinal context, followed by *Helleborus odorus*, albeit to a lesser extent. Owing to the potency and complexity of their root extracts, hellebores have been routinely used in traditional and folk remedies, with subsequent preparations exhibiting cathartic, anthelmintic, and other beneficial properties; however, adverse and toxic potential have been reported [3]. Mass spectrometry and nuclear magnetic resonance profiling of different *Helleborus* species indicate that this genus is a rich source of bioactive compounds: glycosides, saponins, ecdysteroids, and lactones, amongst others [4,5,6,7,8]. Over time, several studies have demonstrated that these hellebore-derived compounds exert a high biological activity on numerous cell types, atypically exerting immunostimulatory and anti-inflammatory effects [9,10,11,12,13,14].

Of particular interest, *Helleborus* extracts have been shown to influence the phenotype of the cells associated with the vascular system, with lasting vasoactive effects [15,16]. In vivo trials have reported the safety profile of administered *Helleborus*-derived compounds to the vascular system, in addition to showing treatment-influenced vascular health. In vivo studies have observed a reduction in atherosclerotic plaque size following treatment with hellebore-derived compounds [17], though the exact mechanism is currently unknown. Atherosclerosis is a multifactorial disease driven by the interplay of several cellular activation states within the vascular wall, of which, *Helleborus* extracts have been shown to influence. For example, several species of the *Helleborus* genus have been reported to influence the activity of several enzymes central to atherogenesis; for example, cyclooxygenase-1 and -2, and 5-lipooxygenase [13]. Moreover, Helleborus-derived extracts have also been shown to reduce the expression of cell surface receptors responsible for immune cell recruitment on endothelial cells, effectively reducing immune cell activity and migration [17]. In addition, several species of *Helleborus* have been shown to demonstrate radical scavenging abilities. Owing to their influence in cardiovascular physiology, the ability of *Helleborus* to target reactive oxygen species (ROS) and interfere in ROS-driven cellular behaviours offers further credence to its application and efficacy in treating vascular-associated disease states. Several other studies have highlighted this antioxidant activity in other genera [18,19,20], and as such, the multifactorial ability of *Helleborus* extracts to exert a therapeutic effect on cells of the vascular system has garnered attention for treating not only disorders of the vascular system, but also vascular-related chronic diseases such as diabetes, cancer, and age-associated degenerative disorders [21].

Despite this body of evidence supporting the use of *Helleborus*-derived treatments in vascular disease states, there are several contrasting reports highlighting the significant risks that *Helleborus*-derived extracts pose as treatments, owing to their purported adverse and toxic properties. Studies have shown preparations of specific species of the *Helleborus* genus to exert potent cytotoxic effects at particular concentration ranges [8,22], with a number of case studies detailing the toxicity and side effects of *Helleborus* administration in experimental setups utilising human biopsies as models of disease [23]. These effects have been shown to extend to and include cells of the vascular system, with *Helleborus*-derived extracts shown to induce anti-proliferative, anti-angiogenic, and pro-apoptotic effects within certain concentration ranges [24].

Interestingly, the aforementioned cytotoxic activities of *Hellebores* were seen to be potentiated in cells which are oncogenic in nature, with primary cells demonstrating increased tolerance to these effects [22]. As such, these results have only broadened their application, with them being utilised as an alternative approach to cancer treatment. For example, the anti-angiogenic effects of particular *Helleborus* extracts may explain their success in treating certain forms of cancer, with studies demonstrating that *Helleborus* treatment inhibits the action of the pro-tumorigenic VEGF-A [24]. Moreover, different genera at different concentrations have shown the ability of *Helleborus* to interfere with the cell-cycle and microtubule dynamics in experimental models of cancer. These anti-proliferative and anti-migratory properties have also been observed in cell models of renal, brain, and breast cancers, amongst many others [19,24,25,26]. Moreover, the translational value of these anti-oncogenic effects is corroborated in clinical data in sporadic patient cases in the literature [9,27]. Taken together, further research on the contrasting biological effects of *Helleborus* and its compounds on models of human physiology is required in order to advance the understanding of its true therapeutic potential.

In this study, we focused on the phytochemical characterisation of *Helleborus odorus* subsp. *cyclophyllus*, combining biochemical assays and chromatographic characterisation to profile the bioactive properties of the extracts derived from the species. The toxicity profile of the extract was then examined in bacterial and mammalian cell cultures, with a focus on a selected number of cell characteristics examined in primary mammalian cultures. Finally, the therapeutic potential of the extract was assessed in a model of the human vasculature in a number of pro-inflammatory disease contexts.

## 2. Materials and Methods

### 2.1. Preparation and Extraction of Helleborus odorus Plant Tissues

*H. odorus* subsp. *cyclophyllus* (A. Brawn Maire and Petitm) mother plants were harvested from the Greek flora (39°22.199′ N, 021°14.109′–110′ E, Altitude: 861–862 m) in spring and subsequently submitted to clonisation at the Holly and Great Monastery of Vatopaidi in Mount Athos, Greece. As a result, certified *H. odorus* subsp. *cyclophyllus* pharmaceutical plant clones were developed. The *H. odorus* subsp. *cyclophyllus* ‘Vatopaidi’ (Italia Herbarium depository number: 2017.012 *Helleborus οdorus* Waldst. & Kit. subsp. *cyclophyllus* A. Brawn Maire and Petitm) was used as experimental material. Air-dried roots from *H. odorus* subsp. *cyclophyllus* were ground using an electrical grinder. Samples (25 mg) were extracted in 1 mL sterile phosphate-buffered saline (PBS) or water on a rocker plate overnight. The extracts were purified by centrifugation (14,000× *g*, room temperature for 5 min) or sterile filtration (for cell and microbial culturing experiments), and the resulting homogenate was collected and analysed immediately.

### 2.2. Phytochemical and Antioxidant Characterisation of Helleborus Extract

The phytochemical content of aqueous *Helleborus* extracts was assessed with biochemical assays following our novel multiparametric protocol [28]. As blanks, PBS diluted with water was used in place of extracts, and each extract was assessed with three replicates.

Polyphenols were quantified by the Folin reagent. Briefly, 100 μL of appropriately diluted extract was mixed with 100 μL of Folin reagent and 100 μL of Na_2_CO_3_ (1.89 M). The mixtures were incubated for 40 min at room temperature, and the absorbance was measured at 765 nm and expressed as equivalents of gallic acid.

Flavonoids were quantified by their reaction with aluminium trichloride. Appropriately diluted extract (100 μL) was mixed with 50 μL of aqueous NaNO_2_ (2%, *w*/*v*) and incubated at room temperature for 10 min. Subsequently, 50 μL of aqueous AlCl_3_ (7.5%, *w*/*v*) and 50 μL of NaOH (3.5 N) were added, and then the mixtures were incubated for 10 min at room temperature. The absorbance was measured at 500 nm and expressed as equivalents of catechin.

Tannins were quantified by their reaction with vanillin under acidic conditions [29]. An amount of 100 μL of appropriately diluted extract was mixed with 100 μL of 4% vanillin in methanol and 50 μL of 100% H_2_SO_4_ and incubated at room temperature for 10 min. Absorbances were measured at 500 nm and expressed as equivalents of catechin.

The metal chelating potential of *Helleborus* extract was assessed for iron and copper ions. The ferric reducing power (FeRP) was determined by the reduction of ferric to ferrous ions, which then react with 2,4,6-tri-pyridyl-s-triazine (TPTZ) to form an absorbing complex at 595 nm. An amount of 100 μL of appropriately diluted extract was mixed with 100 μL of FeRP reagent (250 mM of acetic acid, 1 mM of TPTZ, 0.054% of FeCl_3_·6H_2_O), and then prepared and incubated at room temperature for 40 min. The absorbance was measured at 595 nm and converted to equivalents of gallic acid. The cupric reducing power (CuRP) was determined by the complex of copper ions with neocuproine reagent [bis(neocuproine)copper(II)], which, when reduced by phytochemicals, absorbs at 450 nm. An amount of 125 μL of appropriately diluted extract was mixed with 125 μL of Cu–neocuproine–ammonium acetate reagent (3.33 mM of CuSO_4_·5H_2_O, 2 mM of neocuproine, and 0.33 M of ammonium acetate) and incubated for 40 min at room temperature. The absorbance was measured at 450 nm and converted to equivalents of gallic acid.

The radical scavenging potential of *Helleborus* extracts was assessed for ABTS^•+^, DPPH and galvinoxyl radicals. ABTS^•+^ radical was synthesised by the reaction of 14 mM ABTS and 5 mM potassium persulfate overnight. For the assay, 125 μL of appropriately diluted extract was mixed with 125 μL of ABTS radical cation (ABTS^•+^), which was appropriately diluted in ddH_2_O and incubated at room temperature for 40 min, and the absorbance was then measured at 734 nm. For the DPPH radical, the reagent was diluted in methanol, and 200 μL of DPPH radical reagent was mixed with 50 μL of appropriately diluted extract and incubated in the dark at room temperature for 10 min, and the absorbance was then measured at 515 nm. For galvinoxyl radical, the reagent was diluted in methanol, and 200 μL of galvinoxyl radical reagent was mixed with 50 μL of appropriately diluted extract and incubated in the dark at room temperature for 10 min, and the absorbance was then measured at 435 nm. The % radical scavenging was calculated based on the equation 100 × (A_Radical_ − A_Sample_)/A_Radical_ and expressed as nmoles of equivalents of gallic acid from a linear standard curve.

### 2.3. Metabolite Profiling of the Helleborus sp. Extract Employing Gas Chromatography–Electron Impact–Mass Spectrometry (GC/EI/MS)

Complementary to the abovementioned assays, the deconvolution of the metabolite composition of the extract was performed by employing a gas chromatography–electron impact–mass spectrometry analyser [GC/EI/MS; Agilent 6890 N GC platform (Agilent Technologies Inc., Santa Clara, CA, USA), 5973 T mass selective detector (MSD), 7683 autosampler]. The preparation of the dry samples (15 mg) for the GC/MS analysis was performed in a two-step process using a vacuum concentrator (Eppendorf Concentrator Plus, Eppendorf, Hamburg, Germany), as previously described [30,31], following minor modifications. Briefly, for the methoxymation of the samples, an amount of 80 μL of a methoxylamine hydrochloride solution (20 mg mL^−1^ in pyridine) was added, and the resulting solution was thoroughly vortexed and incubated for 2 h at 30 °C in a water bath (Daihan Labtech, Namyangju-Si, Gyeonggi-do, Korea). In the second step, 80 μL of N-Methyl-N-trimethylsilyl-trifluoroacetamide (MSTFA) was added for silylation, and then the samples were incubated for 1.5 h at 37 °C in the water bath. Prior to their transfer to 200 μL glass microinserters (Macherey-Nagel, Dueren, Germany) into 2 mL glass autosampler vials, the resulting solutions were centrifuged using a benchtop centrifuge (5000 rpm, 10 min) to remove any possible debris that could interfere with the analysis. In total, four replications were prepared and, additionally, experimental blanks that had been prepared following the exact same protocol as the samples were analysed, in order to detect features originating from possible contamination. Also, an aliquot (20 μL) of a methanolic ribitol solution (0.2 mg mL^−1^) served as the internal standard for quality control purposes. The annotation of the metabolites was based on mass fragmentation patterns, using the mass spectra library of the National Institute of Standards and Technology, 2024 (NIST 24, Gaithersburg, MD, USA), and/or analytical standards, where available. The analysis of the derivatised extract was performed under previously described analytical conditions [30,31]. Briefly, the extract (1 μL) was injected on a 30 m long column (HP-5MS, Agilent Technologies Inc.) having a diameter of 0.25 mm and film thickness of 0.25 μm, applying a split ratio of 5:1. Helium was used as the carrier gas at a flow rate of 1 mL min^−1^. Full scan mass spectra were acquired over the range of 50–800 Da (4 scans s^−1^), applying positive electron ionisation (70 eV). The injector’s temperature was set at 230 °C, and the oven temperature program was as follows: initial temperature of 70 °C, stable for 5 min, increase to 310 °C (°C min^−1^), and then stable for 1 min. The MS source temperature was 230 °C, and that of the quadrupole was 150 °C.

### 2.4. Antibacterial Activity of Helleborus odorus subsp. cyclophyllus Extracts

The antibacterial activity of *Helleborus odorus* subsp. *cyclophyllus* extracts was assessed based on the calculation of their minimum inhibitory concentration (MIC) in liquid cultures of the model species *Escherichia coli*. *E. coli* was incubated at 37 °C under continuous agitation at 150 rpm in LB broth medium. The bacterial culture was centrifuged at 3000× *g* for 3 min, and the supernatant was removed. The pelleted bacterial cells were re-suspended in sterile PBS and pelleted again by centrifugation at 3000× *g* for 3 min. The supernatant was discarded and the bacteria were re-suspended in sterile PBS, and then the bacteria concentration was measured based on their absorbance at 600 nm (1 A is equal to 8 × 10^8^ cells/mL). For the assessment of the minimum inhibitory concentration, 100 μL of *Helleborus odorus* subsp. *cyclophyllus* extract (prepared in sterile PBS or water and appropriately diluted in sterile PBS or water) was incubated with 100 μL of LB broth containing 10^4^ cells/mL at 37 °C for 24 h. The MIC was evaluated as the minimum concentration of plant extract that inhibits bacterial growth.

### 2.5. Cell Culture Bioassay

Human Aortic Endothelial Cells (HAECs) (Cat. No. C12271, PromoCell, Heidelberg, Germany) obtained from a 23-year-old Caucasian male were cultured in Endothelial Cell Growth Medium MV (Cat. No. C22020, PromoCell, Heidelberg, Germany) containing the following supplements: fetal calf serum (0.05 mL/mL), endothelial cell growth supplement (0.004 mL/mL), epidermal growth factor (10 ng/mL), heparin (90 µg/mL), and hydrocortisone (1 µg/mL). The culture medium was also supplemented with penicillin (100 IU/mL) and streptomycin (100 µg/mL) (Cat. No. P0781, Merck, Dublin, Ireland). HAEC cultures were maintained in a humidified incubator at 37 °C, 5% CO_2,_ and 95% humidity. Passages 6–12 were used for experimental purposes. For experiments, unless otherwise stated, HAECs were grown to confluence in the specified culture dishes before treatment with *Helleborus odorus* subsp. *cyclophyllus* extracts for up to 48 h. HAEC cultures were treated with a *Helleborus odorus* subsp. *cyclophyllus* extract across a concentration range spanning from 50 ng/mL to 1000 ng/mL. In certain experiments, cultures were also treated with vascular endothelial growth factor (VEGF) (50 ng/mL) (Cat. No. SRP3182, Merck, Dublin, Ireland), platelet-derived growth factor (PDGF) (100 ng/mL) (Cat. No. SRP3138, Merck, Dublin, Ireland), D-glucose (30 mM) (Cat. No. G8644, Merck, Dublin, Ireland), or TNF-α (100 ng/mL) (Cat. No. GF023, Merck, Dublin, Ireland). Additional parallel experiments involving HAEC treatments with superoxide dismutase (SOD) (100 U/mL) (Cat. No. S5395, Merck, Dublin, Ireland) or apocynin (APO) (10 mM) (Cat. No. 178385, Merck Millipore, Dublin, Ireland) were pre-treated with the respective treatment at the final concentration 1 h prior to the addition of *Helleborus odorus* subsp. *cyclophyllus* extract.

### 2.6. Crystal Violet Assay

The crystal violet assay was implemented to measure cell viability and cell proliferation. HAECs were seeded at a density of 15,000 cells/well and 3000 cells/well on a 96-well plate for cell viability and cell proliferation, respectively. The following day, the wells were gently washed with 200 µL/well of PBS before fresh culture medium containing the experimental conditions of interest was added. Following treatment, the plate was once again gently washed with 200 µL of PBS before the cells were fixed with 50 µL of 3.7% formaldehyde for 15 min. After removing the formaldehyde, the cells were gently washed with 200 µL of distilled water before 50 µL of a 0.5% crystal violet solution was added to each well. The cells were incubated with the stain for 30 min before being subjected to six washes with distilled water to remove excess dye and then left to air dry overnight. The following day, the crystal violet was extracted from the cells by adding 50 µL of a 2% SDS solution. Following a 30 min incubation on an orbital rotator to assist the extraction process, the absorbance of the plate was read at 570 nm using a Tecan Infinite M200 (Tecan, Manneford, Switzerland).

### 2.7. Dihydroethidium Assay

Cell-derived reactive oxygen species (ROS) levels were measured using dihydroethidium staining as adapted from a previous protocol [32]. Briefly, HAECs were seeded at a density of 15,000 cells/well on a white 96-well plate 24 h prior to the addition of *Helleborus odorus* subsp. *cyclophyllus* extracts. HAECs were labelled with 3 µM DHE (Cat. No. 37291, Merck, Dublin, Ireland) 30 min prior to the completion of each specific time point. Post-treatment, the 96-well plate was analysed using a Tecan Infinite M200 (Tecan, Manneford, Switzerland) with excitation and emission wavelengths set at 560 and 590 nm, respectively. For normalisation purposes, the DHE signal was baseline corrected with respect to viability levels.

### 2.8. Microscopy

Briefly, HAECs were seeded in 6-well dishes and grown to confluence before being treated ± *Helleborus odorus* subsp. *cyclophyllus* extract. An amount of 3 µM of DHE was added to the HAEC cultures 30 min prior to the cessation of the experimental time point. The culture medium was then removed, and the cells were washed three times with PBS before the cells were fixed for 15 min with 3.7% paraformaldehyde. The fixed cells were washed a further three times before a thin layer of distilled water was added to ensure the monolayer remained hydrated. Stained HAECs were imaged on a Nikon Eclipse Ti fluorescent microscope (Nikon, Tokyo, Japan) at 40× magnification, with the exposure time kept constant across all experimental samples. An unstained control was included for all microscopy work.

### 2.9. Permeability Assay

The analysis of HAEC monolayer permeability was performed using a modified version of the transwell method [33]. Briefly, HAECs were seeded at a high density (1 × 10^6^ cells/insert) into hanging transwell cell culture inserts placed within 6-well dishes (Sarstedt AG & Co, Munich, Germany). Cell culture medium was added to the upper (2 mL) and lower (4 mL) chambers of the transwell insert, and the cells were allowed to adhere overnight. The following day, the HAECs within the transwell were then treated with *Helleborus odorus* subsp. *cyclophyllus* extract ± SOD or APO. Untreated and PBS-treated controls were included for all permeability work. Post-treatment, the medium in the upper and lower chambers was replaced with fresh medium, and fluorescein isothiocyanate (FITC)-labelled 40 kDa dextran was added to the upper chamber at a final concentration of 250 µg/mL. Samples from the lower chamber (28 µL) were taken every 30 min, and after 3 h, all samples were diluted to a final volume of 400 µL with medium before being plated in 100 µL volumes in a 96-well white plate. A Tecan Infinite F200 (Tecan, Mannedorf, Switzerland) was used with excitation and emission wavelengths set at 490 nm and 520 nm, respectively. Permeability is presented as the rate of FITC-dextran 40 kDa transendothelial exchange per hour.

## 3. Results and Discussion

### 3.1. Phytochemical and Antioxidant Properties of Helleborus odorus subsp. cyclophyllus Extract

The initial characterisation of the major categories of phytochemicals and the antioxidant properties of *Helleborus odorus* subsp. *cyclophyllus* was assessed in water and PBS extracts for comparisons (Table 1). Although there are differences in the two preparations, each extract contains several forms of flavonoids, polyphenols, and tannins that are responsible for the radical scavenging potential for ABTS, DPPH, and galvinoxyl radicals and their metal reducing power against iron and copper ions.

### 3.2. GC/EI/MS Metabolite Profiling Revealed the High Content of Bioactive Fatty Acids

**Figure 1:**
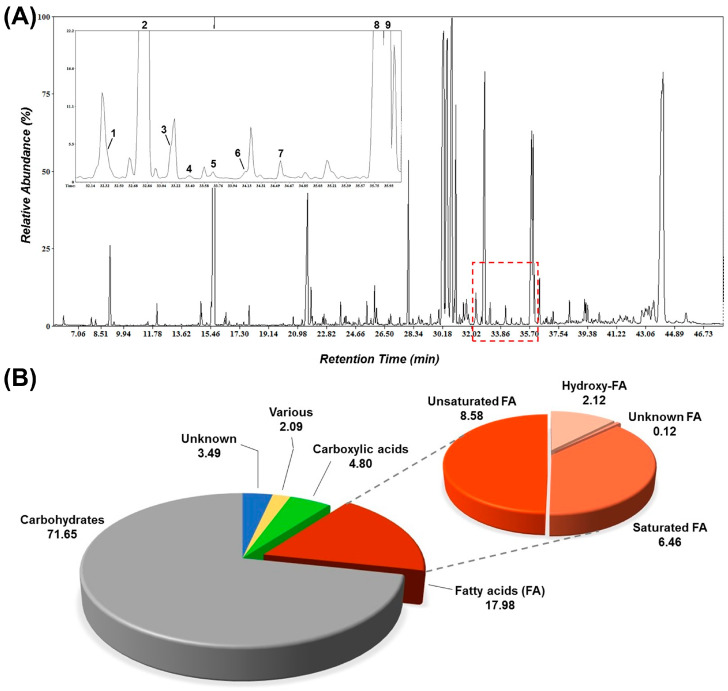
Representative GC/EI/MS total ion chromatogram (TIC) of the analysed *Helleborus odorus* subsp. *cyclophyllus* extract. Annotations for the selected fatty acids are displayed: 1. palmitoleic acid; 2. palmitic acid; 3. 2-hydroxysebacic acid; 4. traumatic acid; 5. methyl oleate; 6. cis-10-heptadecenoic acid; 7. margaric acid; 8. linoleic acid; and 9. oleic acid (**A**). Pie charts displaying the relative abundance (%) of the major chemical groups of the annotated metabolites (**B**).

**Figure 2:**
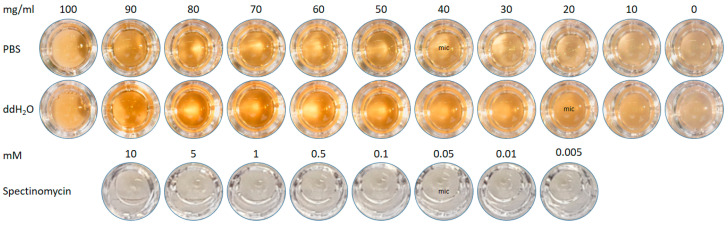
Antimicrobial properties of *Helleborus* extracts in PBS or water against *E. coli* defined by the minimum inhibitory concentration.

The applied GC/EI/MS metabolite profiling protocol resulted in an improved chromatographic separation, which was confirmed by the quality (e.g., number and shape of peaks, baseline) of the obtained total ion chromatograms (Figure 1A). A total of 169 metabolite features, identified at different identification levels, were recorded. The majority of the annotated metabolite features belong to carbohydrates, carboxylic acids, and fatty acids (FAs) (Figure 1B). Interestingly, the extracts had a high relative content in FAs (17.98%), with the most abundant group being the unsaturated FAs, followed by the saturated and hydroxy FAs (Figure 1B and Figure 2 and Supplementary Table S1). Since the phenolic content of the plant has been studied [34], here, the main focus was the deconvolution of its FA content and the correlation to its bioactivity.

FAs are abundant metabolites in plant tissues exhibiting well-established bioactivities, including, among others, antimicrobial, antioxidant, and plant defence-inducing capacity [35,36]. Additionally, they are metabolites of high energetic status that can regulate redox homeostasis via their interference with ROS metabolism [27]. Early evidence has supported the antioxidant capacities of several of the annotated FAs in the analysed *Helleborus odorus* subsp. *cyclophyllus* extract (e.g., myristic acid, palmitic acid), suggesting a positive correlation between the antioxidant activity of the saturated FAs and the increasing chain length [36]. The results also confirmed the superior antioxidant capacity of the majority of the unsaturated FAs being evaluated. Oleic and linoleic acids were the two most abundant unsaturated FAs of the extract (3.59% and 3.30%, respectively), which exhibit superior antioxidant and anticancer profiles [37]. They are also well-known for their anticancer, anti-inflammatory, and immune system-regulating properties [38].

Hydroxy FAs are also metabolites with well-studied bioactivities [27,39]. Among the hydroxy FAs of the extract, azelaic acid was the most abundant (3.44%). The metabolite has established anti-inflammatory and antioxidant activities, as well as antimicrobial activity against Gram-negative and Gram-positive bacteria [39,40,41]. The latter has been attributed to alterations in the intracellular pH [40]. Additionally, azelaic acid exhibits cytotoxicity and anti-proliferative activity against the human malignant melanocyte [42]. Traumatic acid is a metabolite involved in plant responses to stresses that has been reported to protect cells from peroxidation and ROS-induced toxicity [27].

Based on the obtained evidence and the information from the literature, the observed bioactivities of the extract cannot be attributed to a single component. In conclusion, it is plausible to suggest that the recorded bioactivity of the analysed extract can be, at least partially, attributed to its FA content as a whole, an observation that is in line with previous studies on similar FA-rich sources and their potential for applications in the agrochemical, food, and pharmaceutical industries [43,44].

### 3.3. Antimicrobial Properties of Helleborus odorus subsp. cyclophyllus Extract

*Helleborus* extracts in sterile PBS or water were assessed by the minimum inhibitory concentration (MIC) in *E. coli* cultures (Figure 2). The MIC was verified by growth as 40 mg/mL and 20 mg/mL in PBS and water, respectively, and compared with spectinomycin as a positive control with a MIC value of 0.05 mM. The use of *Helleborus* preparations to treat infectious diseases is an established practise [10,15], and while research has examined the impact of *Helleborus* preparations on physiological systems, the data on the specific antimicrobial activity of *Helleborus* in the context of these infectious diseases is limited in comparison. Rosselli [45] demonstrated the antibacterial influence of *Helleborus bocconei* extracts against several strains of pathophysiological microorganisms, with later work by Puglisi [46] demonstrating similar effects with a specific focus on strains known to promote respiratory infections. Interestingly, Rosselli reported an MIC in the µg/mL range for *Helleborus bocconei* in all microorganisms examined, including *E. coli*. However, Puglisi reported an MIC in the mg/mL range for extracts from the same *Helleborus* species against *E. coli.* Our data agrees with Puglisi in that *Helleborus odorus* displays an MIC in the mg/mL range, although the extraction methods of the plant materials differ.

### 3.4. Helleborus Concentrations Greater than 50 ng/mL Dose- and Time-Dependently Reduce HAEC Viability

**Figure 3:**
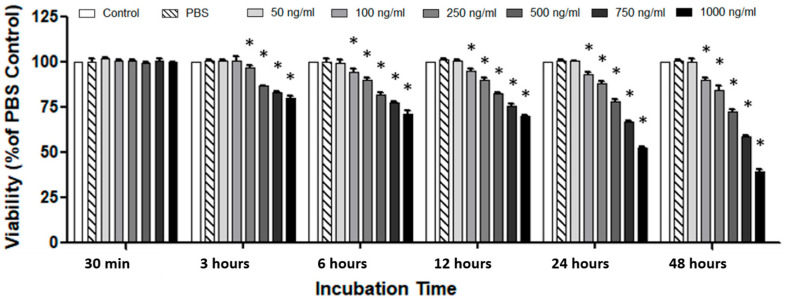
The effect of *Helleborus* concentration on HAEC viability (N = 3). HAECs were treated with *Helleborus* (50 ng/mL–1000 ng/mL) for up to 48 h. Crystal violet stain was added at the end of the incubation period in order to quantify cell density of the HAECs following *Helleborus* treatment. * *p* ≤ 0.05 versus the untreated control.

In order to investigate the potential physiological impact of *Helleborus odorus* subsp. *cyclophyllus*, HAECs were used as a model of the vascular system, and the impact of dose and treatment time on cell viability was first examined by crystal violet assay. Treatment of HAECs with *Helleborus* extracts (50 ng/mL–1000 ng/mL) dose- and time-dependently reduced HAEC viability up to 48 h. Concentrations of ≤50 ng/mL had no significant impact on cell viability (Figure 3).

Several studies on *Helleborus*-derived extracts have revealed that they, or metabolites from such, demonstrate cytotoxic activity. Lindholm [47] found in a screening of 100 plant extracts that *Helleborus cyclophyllus* and *Helleborus caucasicus* both demonstrated potent antitumoral activity in vitro. Similar studies of *Helleborus niger* [23], *Helleborus bocconei* [48], *Helleborus multifidus* [19], and *Helleborus purpurascens* [49,50] demonstrated similar cytotoxic potential in cancer cell lines, the efficacy of which has seen patents granted for use of *Helleborus* extracts as cytotoxic agents, with specific application of *Helleborus* derivative as an anticancer agent, owing to the reported antitumor activity in the nanomolar range [51].

### 3.5. Helleborus-Dependent ROS Induction Influences HAEC Viability

**Figure 4:**
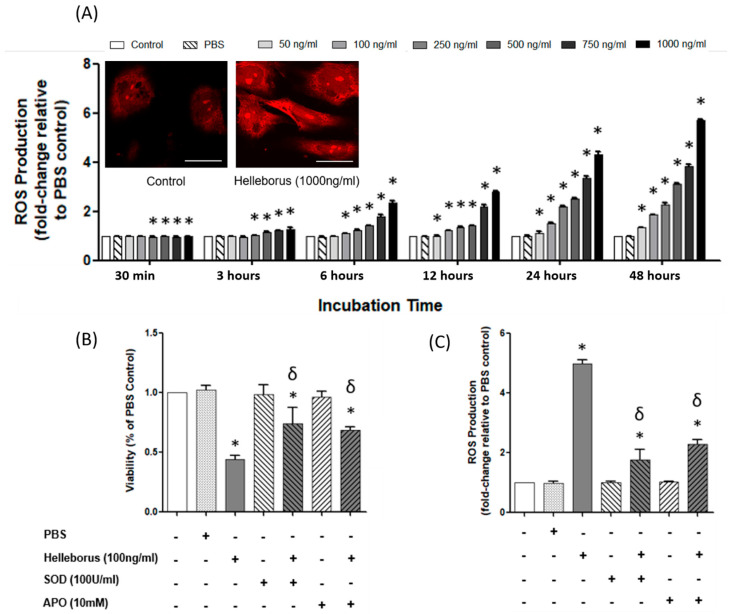
The effect of *Helleborus* concentration on ROS induction in HAECs (N = 3). HAECs were treated with *Helleborus odorus* subsp. *cyclophyllus* extract (50 ng/mL–1000 ng/mL) for up to 48 h. DHE stain (3 µM) was added 30 min prior to the end of the incubation period to quantify the production of ROS in HAECs. (**A**) The time- and dose-dependent impact of *Helleborus odorus* subsp. *cyclophyllus* on ROS production in HAECs. All values are baseline corrected to cell viability. Microscopic visualisation of ROS in HAECs after 48 h of treatment ± 1000 ng/mL of *Helleborus odorus* subsp. *cyclophyllus* extract (insert). Red: DHE staining for ROS. Scale bar is 30 µm. Images are at 40× and are representative. (**B**) The effect of SOD and APO individually on *Helleborus*-induced ROS. (**C**) The effect of SOD and APO individually on *Helleborus*-induced cell death. * *p* ≤ 0.05 versus the untreated control; δ *p* ≤ 0.05 versus the *Helleborus*-treated cultures (100 ng/mL).

The impact of *Helleborus* treatment of HAECs on ROS induction was investigated quantitatively using a dihydroethidium assay. Treatment of HAECs with *Helleborus* extracts (50 ng/mL–1000 ng/mL) dose- and time-dependently significantly induced ROS production (Figure 4A). The conversion of dihydroethidium to ethidium bromide courtesy of ROS induction was confirmed by fluorescence microscopy (Figure 4 insert). To focus on the potential non-lethal effects of *Helleborus odorus* subsp. *cyclophyllus*, all subsequent experiments focused on a narrow range of concentrations (50/100/250 ng/mL). Co-treatment of HAECs treated with *Helleborus* (50/100/250 ng/mL) with SOD (100 U/mL) or APO (10 mM) was shown to significantly attenuate the effects of *Helleborus* treatment on cell viability (Figure 4B) through the significant amelioration of ROS (Figure 4C).

Previous studies have reported contrasting data on the antioxidant potential of *Helleborus* species. Work by Apetrei et al. [18] and Păun-Roman et al. [20] revealed that extracts of *Helleborus purpurascens* demonstrated potent antioxidant potential in vitro, with other works on material/s isolated from *Helleborus multifidus*, *Helleborus hercegovinus*, and *Helleborus odorus* demonstrating similar abilities. A study by Malik [13], however, reported that *Helleborus purpurascens* had negligible antioxidant activity, with similar results reported for *Helleborus niger* and *Helleborus odorus*. While our biochemical data suggests that extracts of *Helleborus odorus* do contain compounds of antioxidant potential (Figure 1), other compounds that are extracted appear to induce pro-oxidant pathways and/or interfere with the ability of the antioxidant compounds to exert their effect on HAEC cultures. Activation of NADPH oxidase has previously been shown to be responsible for pro-inflammatory induction of ROS in endothelial cells [52]. Ablation of ROS using APO in our data indicates that extracts of *Helleborus odorus* promote assembly and activation of the NADPH oxidase complex, promoting a pro-inflammatory phenotype in HAECs as a result.

### 3.6. Helleborus-Dependent ROS Induction Influences HAEC Permeability

**Figure 5:**
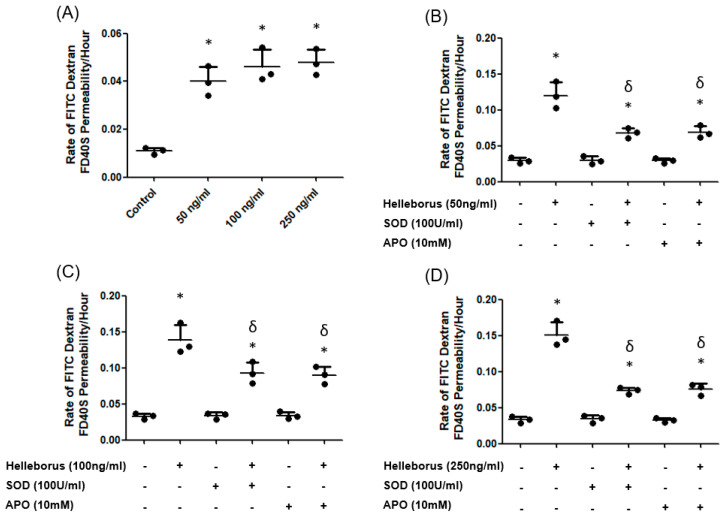
The effect of *Helleborus* concentration on HAEC permeability (N = 3). HAECs were treated for up to 48 h with *Helleborus* (50, 100 or 250 ng/mL) in the absence (**A**) and presence (**B**–**D**) of either superoxide dismutase (SOD, 100 U/mL) or apocynin (APO, 10 mM). HAEC barrier integrity was then monitored by transendothelial permeability assay with data represented as the mean rate of FITC-dextran diffusion: %FD40 TEE.hour^−1^. * *p* ≤ 0.05 versus the untreated control; δ *p* ≤ 0.05 versus the *Helleborus*-treated cultures.

The impact of *Helleborus* treatment (50/100/250 ng/mL) on HAEC permeability was examined using a transwell permeability assay. Treatment of HAECs with *Helleborus* (50/100/250 ng/mL) saw a significant increase in monolayer permeability (Figure 5A). Co-treatment of *Helleborus*-treated HAECs with SOD (100 U/mL) or APO (10 mM) was shown to significantly attenuate the *Helleborus*-induced increase in monolayer permeability for 50 (Figure 5B), 100 (Figure 5C), and 250 ng/mL (Figure 5D) of *Helleborus* extract up to 48 h of treatment.

Previous data has shown compounds derived from *Helleborus* to be cardioprotective, owing to its ability to directly influence the endothelium. Protoanemonin, a γ-lactone that can be isolated from members of the *Helleborus* family, has been shown to modulate endothelial function and promote vascular integrity. Work by Hu [53] and Duan [54] showed that anemonin, a secondary metabolite of protoanemonin, downregulates the expression of the key signalling molecules inducible nitric oxide synthase (iNOS) and endothelin-1 (ET-1) in microvascular endothelial cells. In nature, pleiotropic molecules decrease the expression of both proteins, in parallel with intercellular adhesion molecule-1 (ICAM-1), which promotes increased endothelial barrier function, in addition to an anti-inflammatory phenotype. Our contrasting data suggests that extracts of *Helleborus odorus* subsp. *cyclophyllus* disrupt endothelial barrier integrity, partly through a ROS-mediated pro-inflammatory mechanism. Previous data from our laboratory has demonstrated that NADPH oxidase plays an influential role in pro-inflammatory disruption of the endothelial barrier [33,55], with the protective effects of APO indicating a similar trend in response to *Helleborus odorus* treatment.

### 3.7. Helleborus Treatment Attenuates VEGF and PDGF Pro-Angiogenic Effects

**Figure 6:**
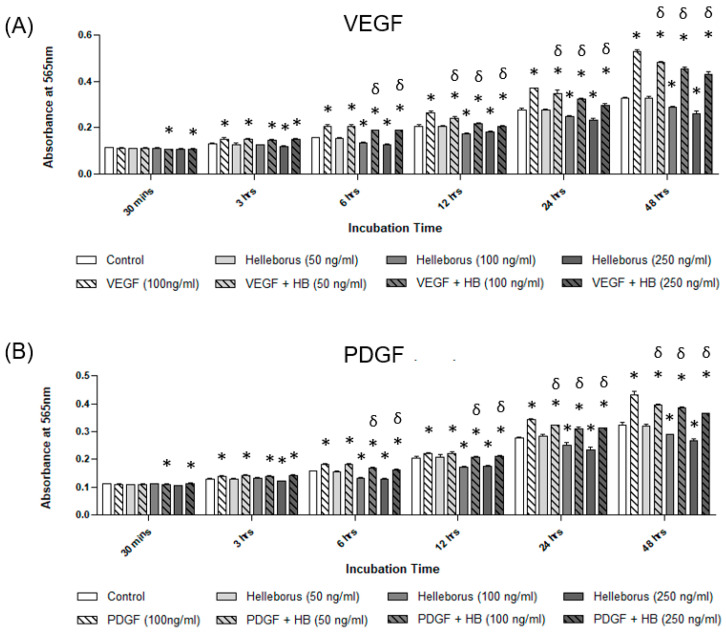
The effect of *Helleborus* treatment on pro-angiogenic stimulation (N = 3). HAECs were treated for up to 48 h with VEGF (50 ng/mL) (**A**) or PDGF (100 ng/mL) (**B**). The HAEC cell number was then monitored by crystal violet staining, with data represented as the absorbance at 565 nm. * *p* ≤ 0.05 versus the untreated control; δ *p* ≤ 0.05 versus the (**A**) VEGF- or (**B**) PDGF-treated cultures.

The impact of *Helleborus* treatment (50/100/250 ng/mL) on pro-angiogenic conditions was examined using a crystal violet assay adapted for measuring proliferation. Treatment of HAECS with VEGF (50 ng/mL) (Figure 6A) or PDGF (100 ng/mL) (Figure 6B) saw a significant increase in cell number when compared to untreated controls. Co-treatment of VEGF- or PDGF-treated HAECs with *Helleborus odorus* subsp. *cyclophyllus* extract showed a dose-dependent reduction in the proliferative effects of both VEGF and PDGF, with significant effects observed across all concentrations (50/100/250 ng/mL) from 12 h for VEGF and 24 h for PDGF.

### 3.8. Helleborus Treatment Exacerbates Hyperglycaemic and TNF-α Pro-Apoptotic Effects

**Figure 7:**
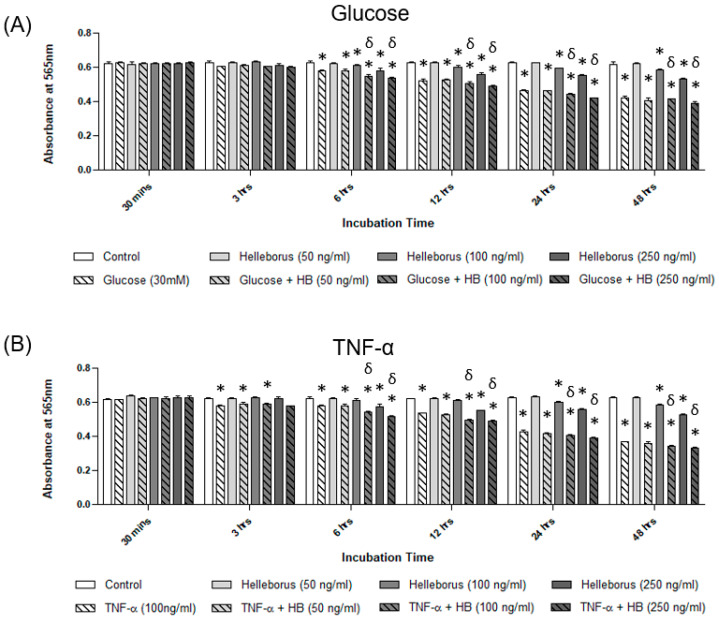
The effect of *Helleborus* treatment on pro-apoptotic stimulation (N = 3). HAECs were treated for up to 48 h with glucose (30 mM) (**A**) or TNF-α (100 ng/mL) (**B**). The HAEC cell number was then monitored by crystal violet staining, with data represented as the absorbance at 565 nm. * *p* ≤ 0.05 versus the untreated control; δ *p* ≤ 0.05 versus the (**A**) glucose- or (**B**) TNF-α treated cultures.

The impact of *Helleborus* treatment (50/100/250 ng/mL) on pro-apoptotic conditions was examined using a crystal violet assay. Treatment of HAECs with D-glucose (30 mM) (Figure 7A) or TNF-α (100 ng/mL) (Figure 7A) showed a decrease in cell number when compared to untreated controls. Co-treatment of hyperglycaemic- or TNF-α-treated HAECs with *Helleborus* extract saw a dose-dependent exacerbation in the pro-apoptotic effects of both D-glucose and TNF-α, with significant effects observed for 100 ng/mL and 250 ng/mL of *Helleborus* treatment from 6 h of treatment.

In a screening of 25 medicinal plant extracts, Malik [13] reported that *Helleborus purpurascens* was the best at inhibiting the pro-inflammatory enzymes COX-1, COX-2, and 5-LOX, with *Helleborus niger* and *Helleborus odorus* also exhibiting similar, albeit less potent, functionality. Subsequent GC-MS analysis of *Helleborus purpurascens* fractions revealed that the fraction is comprised of a complex of fatty acids, of which α-linoleic acid was the predominant constituent, demonstrating the greatest inhibitory action towards these enzymes. In addition to our findings of antioxidant potential (Table 1), our GC/EI/MS analysis reported relatively high levels of linoleic acid in the *Helleborus odorus* subsp. *cyclophyllus* extracts used in this manuscript and should therefore promote an anti-inflammatory phenotype. To evaluate the therapeutic efficacy of extracts of *Helleborus odorus* subsp. *cyclophyllus*, our data firmly suggests that the extracts exacerbate the pro-inflammatory effects induced by the agonists employed.

Previous examinations of the therapeutic potential of *Helleborus* extracts agree with these findings. In addition to reporting negligible antioxidant activity, Čakar et al. [19] also found the *Helleborus* extracts to exhibit anti-proliferative properties, which is a finding that several other screenings of *Helleborus*-derived compounds have corroborated, in addition to cytotoxic activity. Data pertaining to the vasculature is limited; however, a study by Felenda [24] also reported anti-proliferative and anti-angiogenic effects in *Helleborus niger*-treated HUVECs.

Similar extracts from other species (*Helleborus niger*) exhibit cytotoxicity to cancer and leukaemia cell lines and primary cells of patients with childhood ALL and AML, as they showed inhibition of proliferation by induction of apoptosis [23]. In addition, extracts have been shown to have differential cytotoxicity towards tumour cell lines and healthy human T- and NK-cells by inhibiting proliferation and inducing apoptosis [56]. Recently, another species (*Helleborus cyclophyllus* Boiss) induced apoptotic cell death and vesicular formations on A549 human bronchial epithelial adenocarcinoma cells selectively and in comparison to normal cells, which were not affected [22]. Although these are in vitro studies and can reach as far as they are limited to a translational model, single-patient case studies have been reported in which extracts of hellebores have been used instead of, or in conjunction with, cancer treatment. In a recent case, a minor regression and long-time survival were observed in a cancer patient who did not receive any other cancer treatment against malignant pleural mesothelioma. The patient remained in good health, and death occurred 56 months after the initial diagnosis [57].

## 4. Conclusions

The genus *Helleborus* has demonstrated a diverse range of physiological abilities, many of which have proven beneficial therapeutic actions that are unique and/or contrasting with respect to each species and subspecies. However, *Helleborus* species remain relatively unexplored, particularly at the cellular level, and investigation of each species and its inherent profile of chemical compounds could potentially identify bioactive molecules of therapeutic potential. As such, the examination of species of the *Helleborus* genus warrants further investigation, and in this study, *Helleborus* extracts were found to contain several phytochemical compounds and exhibited antioxidant, antimicrobial, and cytotoxic potential. Given their cytotoxic and antibacterial action, *Helleborus* extracts have potential as alternative approaches to chemotherapy, as evidenced in case studies highlighting their importance in medicine. Further examination with in vitro and in vivo models would provide important insight into the compound-specific properties of the constituents of the extracts and potentially yield new bioactive molecules to be isolated and investigated further.

## Figures and Tables

**Table 1:** Phytochemical and antioxidant properties of *Helleborus odorus* subsp. *cyclophyllus* extracts.

Extract	Polyphenols	Flavonoids	Tannins	FeRP	CuRP	ABTS^•+^	DPPH	Galvinoxyl
PBS	3.58 ± 0.25	0.715 ± 0.02	8.55 ± 0.82	27.8 ± 0.8	21.2 ± 0.8	8.05 ± 0.8	1.05 ± 0.028	7.94 ± 0.64
Water	2.96 ± 0.47	**0.135 ± 0.033**	9.97 ± 0.87	**42.8 ± 1.7**	**17.8 ± 0.9**	**4.3 ± 0.16**	**0.995 ± 0.0165**	**3.62 ± 0.78**

Data represent average ± SD (N = 3) and were considered statistically significant (bold font) by Student’s *t*-test between extractions. Polyphenols, metal chelation potential, and the % of radical scavenging were expressed as equivalents of gallic acid per g of extract, whereas flavonoids and tannins were expressed as equivalents of catechin per g of *Helleborus* dried root sample.

## Data Availability

The datasets used and/or analysed during the current study are available from the corresponding author upon reasonable request. The original contributions presented in this study are included in the Supplementary Materials.

## Supplementary Materials

The following supporting information can be downloaded at: https://www.mdpi.com/article/10.3390/biology15110852/s1. Supplementary Table S1: Fatty acid content of the analysed *Helleborus* sp. extract. Information was retrieved from the repositories of NIST, PubChem, KEGG, and LipidMaps.

## Abbreviations

The following abbreviations are used in this manuscript:

ALL	Acute lymphocytic leukaemia
AML	Acute myeloid leukaemia
APO	Apocynin
COX	Cyclooxygenase
DHE	Dihydroethidium
ET-1	Endothelin-1
FA	Fatty acid
FITC	Fluorescein isothiocyanate
HAECs	Human aortic endothelial cells
ICAM-1	Intercellular adhesion molecule-1
iNOS	Inducible nitric oxide synthase
LOX	Lipoxygenase
MIC	Minimum inhibitory concentration
PBS	Phosphate-buffered saline
PDGF	Platelet-derived growth factor
ROS	Reactive oxygen species
SOD	Superoxide dismutase
TNF-α	Tumour necrosis factor α
VEGF	Vascular endothelial growth factor
