# Supplementary material for: Helleborus odorus subsp. cyclophyllus: An Unexploited Source of Antioxidant, Antimicrobial, and Cytotoxic Bioactivity
Source: Biology (Basel). 2026 May 29;15(11):852. doi: 10.3390/biology15110852 (PMC13255700; doi:10.3390/biology15110852)
Supplement: Supplementary file 1 [file biology-15-00852-s001.zip › Supplementary Table S1.pdf]

**Supplementary Table 1.** Fatty acid content of the analyzed *Helleborus* sp. extract. Information was retrieved from the repositories of NIST, PubChem, KEGG, and LipidMaps.

| Quant mass (*m/z*) | Metabolite name | Metabolite derivative | Synonym(s) | PubChem (CID) | LIPIDMAPS | CAS Registry Number | Molecular Formula | Relative Composition (%) |
|---|---|---|---|---|---|---|---|---|
| ***Hydroxy Fatty Acids*** | | | | | | | | |
| 317 | 2-Hydroxysebacic acid (C10:1;O3) | 3TMS | 3-Hydroxy-decanedioic acid | 3017884 | LMFA01170092 | 73141-46-5 | C19H38O4 | 0.07 |
| 317 | 2-Hydroxysebacic acid (C10:1;O3) | 2TMS | 3-Hydroxy-decanedioic acid | 3017884 | LMFA01170092 | 73141-46-5 | C19H38O4 | 0.39 |
| 179 | 3-Hydroxy valeric acid (C5:0;O) | 2TMS | 3-Hydroxy-pentanoic acid | 107802 | LMFA01050008 | 10237-77-1 | C5H10O3 | 0.03 |
| 173 | 3-Hydroxyoctanoic acid (C8:0;O) | 1TMS | 3-Hydroxyoctanoate | 26613 | LMFA01050314 | 14292-27-4 | C8H16O3 | 0.10 |
| 173 | 7-Hydroxyheptanoic acid (C7:0;O) | 2TMS | 7-hydroxy-heptanoic acid | 138016 | LMFA01050019 | 3710-42-7 | C7H14O3 | 0.76 |
| 289 | 7-Hydroxyoctanoic acid (C8:0;O) | 2TMS | 7-Hydroxyoctanoate | 5312863 | LMFA01050316 | 17173-14-7 | C8H16O3 | 0.12 |
| 317 | Azelaic acid (C9:1;O2) | 2TMS | Nonanedioic acid | 2266 | LMFA01170054 | 123-99-9 | C9H16O4 | 3.44 |
| 331 | Sebacic acid (C10:1;O2) | 2TMS | Decanedioic acid | 5192 | LMFA01170006 | 111-20-6 | C10H18O4 | 0.26 |
| 187 | Suberic acid (C8:1;O2) | 1TMS | 1,8-Octanedioic acid; Octanedioic acid | 10457 | LMFA01170001 | 505-48-6 | C8H14O4 | 0.35 |
| 73 | Traumatic acid (C12:2;O2) | 2TMS | (2E)-Dodecenedioic acid; Dodec-2-enedioic acid | 5283028 | LMFA01170002 | 6402-36-4 | C12H20O4 | 0.05 |
| 229 | Undecanedioic acid (C11:1;O2) | 2TMS | 1,9-Nonanedicarboxylic acid | 15816 | LMFA01170007 | 15816 | C11H20O4 | 0.05 |
| | | | | | | | ***Total Hydroxy Fatty Acids*** | ***5.68*** |
| ***Saturated Fatty Acids*** | | | | | | | | |
| 229 | Decanoic acid (C10:0) | 1TMS | n-Capric acid | 2969 | LMFA01010010 | 334-48-5 | C10H20O2 | 0.05 |
| 257 | Dodecanoic acid (C12:0) | 1TMS | Dodecanoic acid; Dodecanoate; | 3893 | LMFA01010012 | 143-07-7 | C12H24O2 | 0.03 |

| | | | | | | | | |
|---|---|---|---|---|---|---|---|---|
| | | | Dodecylcarboxylate; Lauric acid | | | | | |
| 187 | Heptanoic acid (C7:0) | 1TMS | Enanthic acid; Oenanthic acid | 8094 | LMFA01010007 | 111-14-8 | C7H14O2 | 0.76 |
| 173 | Hexanoic acid (C6:0) | 1TMS | Hexanoate; Hexylic acid;n-Caproic acid | 4740 | LMFA01010006 | 14246-15-2 | C6H12O2 | 0.09 |
| 285 | Myristic acid (C14:0) | 1TMS | Tetradecanoic acid; Tetradecanoate | 11005 | LMFA01010014 | 18603-17-3 | C14H28O2 | 0.08 |
| 215 | Nonanoic acid (C9:0) | 1TMS | Nonanoate; Pelargonic acid | 8158 | LMFA01010014 | 112-05-0 | C9H18O2 | 0.08 |
| 201 | Octanoic acid (C7:0) | 1TMS | Caprylic acid; Octylic acid | 8658 | LMFA01010008 | 124-07-2 | C8H16O2 | 0.30 |
| 313 | Palmitic acid (C16:0) | 1TMS | Hexadecanoic acid; Hexadecanoate; Hexadecylic acid; Palmitate; Cetylic acid | 985 | LMFA01010001 | 57-10-3 | C16H32O2 | 1.54 |
| 299 | Pentadecanoic acid (C15:0) | 1TMS | Pentadecylic acid | 13849 | LMFA01010015 | 1002-84-2 | C15H30O2 | 0.40 |
| 341 | Stearic acid (C18:0) | 1TMS | Octadecanoic acid | 5281 | LMFA01010018 | 57-11-4 | C18H36O2 | 0.99 |
| 75 | Valeric acid (C6:0) | 1TMS | Pentanoic acid | 7991 | LMFA01010005 | 109-52-4 | C5H10O2 | 0.47 |
| | | | | | | ***Total Saturated Fatty Acids*** | | ***4.80*** |
| ***Unsaturated Fatty Acids*** | | | | | | | | |
| 185 | 2-Heptenoic acid (C7:1n-5) | 1TMS | 2-Heptenic acid | 5282709 | LMFA01030012 | 18999-28-5 | C7H12O2 | 0.05 |
| 171 | 2-Hexenoic acid (C6:1n-4) | 1TMS | Isohydrosorbic acid | 12467038 | LMFA01030008 | 1577-28-2 | C6H10O2 | 0.01 |
| 199 | 2-Octenoic acid (C8:1n-6) | 1TMS | 3-n-Amyl acrylic acid | 5282713 | LMFA0103001 | 1577-96-4 | C8H14O2 | 0.22 |
| 325 | cis-10-Heptadecenoic acid (C17:1n-7) | 1TMS | 10Z-Heptadecenoic acid | 5312435 | LMFA01030283 | 29743-97-3 | C17H32O2 | 0.03 |
| 337 | Linoleic acid (C18:2n-6,9) | 1TMS | Linoleate; (9Z,12Z)-Octadecadienoic acid; 9-cis,12-cis-Octadecadienoate; 9-cis,12-cis-Octadecadienoic acid | 5280450 | LMFA01030120 | 60-33-3 | C18H32O2 | 3.30 |

| | | | | | | | | |
|---|---|---|---|---|---|---|---|---|
| 327 | Margaric acid (C17:1n-8) | 1TMS | Heptadecanoic acid | 5312440 | LMFA01030060 | 104255-81-4 | C17H32O2 | 0.16 |
| 55 | Methyl oleate (20:1) | 1TMS | Oleic acid methyl ester | 5364509 | LMFA07010880 | 112-62-9 | C19H36O2 | 0.01 |
| 339 | Oleic acid (C18:1n-9) | 1TMS | (9Z)-Octadecenoic acid; (Z)-Octadec-9-enoic acid; Oleate | 445639 | LMFA01030002 | 112-80-1 | C18H34O2 | 3.59 |
| 311 | Palmitoleic acid (C16:1n-7) | 1TMS | (9Z)-Hexadecenoic acid; Palmitoleic acid; cis-9-Hexadecenoic acid; (9Z)-Hexadec-9-enoate | 445638 | LMFA01030056 | 373-49-9 | C16H30O2 | 0.09 |
| | | | | | | | ***Total Unsaturated Fatty Acids*** | ***7.46*** |
| | | | | | | | ***Total Fatty Acids*** | ***17.86*** |
